# Sleeping Spermatozoa: The Symbolism of Gamete Cryopreservation in the Context of Cancer in AYAs

**DOI:** 10.3390/life15050685

**Published:** 2025-04-23

**Authors:** Isabelle Koscinski, Céline Mazzoleni, France Verhaeghe, Jean-Philippe Klein, Célia Ravel

**Affiliations:** 1 Laboratory of Biology of Reproduction, Saint Joseph Hospital, 13008 Marseille, France; cmazzoleni@hopital-saint-joseph.fr (C.M.); france.verh@hotmail.fr (F.V.); 2 Institut National de la Santé et de la Recherche Médicale, Unit 1256, Nutrition-Génétique et Exposition aux Risques Environnementaux, 54505 Nancy, France; 3 Reproductive Biology Department, University Hospital Center of Saint Etienne, 42055 Saint-Etienne, France; jeanphiklein@hotmail.fr; 4 Health, Systemic, Process Laboratory (P2S), UR4129, University Claude Bernard Lyon 1, University of Lyon, 69008 Lyon, France; 5 Laboratory of Biology of Reproduction—CECOS, CHU de Rennes, 35000 Rennes, France; celia.ravel@chu-rennes.fr; 6 Univ Rennes, CHU Rennes, Inserm, EHESP, Irset (Institut de recherche en santé, environnement et travail)-UMR_S 1085, F-35000 Rennes, France

**Keywords:** sperm freezing, cancer, oncofertility, AYAs

## Abstract

For adolescents and young adults (AYAs) with cancer, fertility preservation is recommended before starting gonadotoxic treatments. This is an important aspect of psychological support in the treatment of the disease. However, the enormous psychological impact of this procedure on adolescents and young adults with cancer needs to be addressed by professionals. The traumatic nature of cancer diagnosis disrupts the psychosocial development of AYAs. A young adolescent’s perception of reproduction, and in particular of sperm freezing, is greatly altered by the disease. For a teenager, the success of sperm banking results from a positive balance between facilitators and barriers, which are mentioned here. Moreover, this article proposes a symbolic interpretation of sperm banking, referring to landmarks integrated during childhood, especially in fairytales. Furthermore, it offers an original video documentary that can be used as an information support to help AYAs adhere to the process of preserving their fertility through sperm freezing.

## 1. Introduction

Adolescents and young adults (AYAs) with cancer have specific psychosocial needs, with reproductive health being a major concern [1]. In recent decades, cancer treatments have improved the long-term survival of these young patients. As potential infertility may occur after oncological therapies, fertility preservation is recommended prior to treatments [2]. International recommendations emphasize the importance of consultation with a reproductive specialist to provide comprehensive information. However, sperm cryopreservation makes it possible to desynchronize gamete production and procreation, raising ethical concerns.

In adolescents, specific structural and psychosocial factors can hinder the process of sperm preservation. In this transition period between childhood and adulthood, when exiting from the familial environment, boys take on more and more independence, and disease can appear like a clap of thunder in a clear sky. The announcement of cancer is often experienced as a rupture, isolating the young person from school, leisure activities, and friends. Young people emphasize a loss of normality, a loss of independence, and the fear of physical changes associated with treatment [3]. In fact, treatments and side effects double the physical upheavals experienced and represent a possible weakening of the young person’s main points of reference and support. The relationship with one’s body, one’s self-image, one’s narcissism, one’s confidence in one’s qualities and one’s ability to think, one’s confidence in one’s environment, one’s autonomy [4], and one’s emerging or consolidating sexuality [5] are completely disrupted. Cancer becomes part of the development process and represents a real traumatic crisis due to the rupture it causes, which disrupts the orientation of young patients [6].

Gripped by a sense of fragility and danger, young people seek refuge in what seems the most reassuring to them—the cocoon of the family, where they seek reassurance and containment [7,8]. The family, in turn, is as protective, supportive, and reassuring as possible. One-third of AYAs mention maternal overprotection [9,10]. Even though they generally accept daily help from their parents, young people express the impression of being treated like children and, consequently, a feeling of returning to childhood [11,12]. Moreover, the medical team has a similar attitude.

In order to maintain a certain illusion of autonomy and independence, some AYAs may develop aggressive movements, moments of withdrawal or transgression [13]. For young people with cancer, the desire for separation and independence from their parents conflicts with the desire to be supported by them [11].

However, the explanation of the cancer treatment strategy and its side effects, as well as the fertility preservation procedure, is very technical, with a scientific reason for each action and a very limited space for the human character of the sick patient.

Offering sperm banking to pediatric populations requires continued efforts to facilitate decision making prior to cancer treatment [14]. To find the most appropriate way to support adolescents in the process of sperm banking, it appears crucial to understand all factors implicated in the success of this process. In order to cope with the traumatic situation, young adolescents draw on childhood resources that are not too far away. They find models there, such as fairytale heroes who show how courage is rewarded with happy outcomes from extremely dangerous situations. This article proposes an interpretation of the symbolism of gamete cryopreservation in the context of cancer in AYAs for optimal communication between medical health professionals and patients. This article suggests an original approach to help AYAs regain some self-confidence. We also discuss the issues that parents and AYAs are reluctant to raise but that professionals need to be aware of.

## 2. Meaning of Parenthood for Young Cancer Patients

In adolescence, psychosocial concerns are focused on the present time between a desire for freedom/independence according to physical changes and the world of childhood linked to the family and school. The disease gives a dark color to the present, and the cognitive abilities of adolescents allow them to understand the seriousness of the situation. In this context, there is naturally very little room to consider the distant future. The projection of a future where the young adolescent is cured and wants to start a family is completely abstract. However, the young adolescent associates and confuses fertility and sexuality. Throughout adolescence, healthy growing young people attempt to psychologically appropriate their sexualized bodies. Cancer and the side effects of treatment can be experienced as obstacles to the process of sexualization and progression to adulthood, or as a means of control and defense against the onset of sexual puberty. Later, when treatment is underway, side effects (alopecia, loss of pubic hair, weight loss, muscular atrophy, and soreness of certain erogenous zones due to mucositis) can cause a feeling of erasure of the sexual body, generating a phantasm of returning to an infantile body or even to an asexual body [15]. The desexualization of the body through treatment also has a special place in the Oedipal problem—it can hinder the Oedipal identification of the same-sex parent and produce a phantasm of punishment for the revival of Oedipal and parricidal desires. For these young adolescents, the suggestion of collecting sperm through masturbation may support the process of body sexualization that is disrupted by the onset of cancer but necessary for adulthood. Older adolescents and young adults also often confuse sexuality and masculinity/virility with fertility. Since becoming a parent is a means of increasing self-esteem, the risk of losing this status can be devastating. This feeling is exacerbated by the fear that erasing sexuality from the body will make them less lovable and desirable, and that the danger of death hanging over them will make people reluctant to enter into a lasting relationship with them [15].

For all, the meaning of fertility depends on familial, social, and cultural representations. Cultural worldviews and close relationships may enhance the prospect of achieving literal or symbolic immortality. Indeed, fertility offers symbolic immortality through the transmission of one’s own genes to offspring, as children can carry on the family name and traditions. Children may also reinforce their parents’ belief in the dominant cultural worldview by adopting this culture’s core beliefs and values [16]. One of the difficulties faced by AYAs is that they often do not have a partner to build a parental project with. The question of their fertility may, therefore, seem unclear. However, certain societies may regard parenthood as vital for the long-term strength of the group. This is particularly important in tribal and religious settings. These functions of parenthood have been highlighted empirically in several studies, which showed that the salience of mortality led participants to develop a greater desire to have children [17]. Becoming a father is a potentially dynamic transition that brings many social and psychological benefits, but also some daunting challenges [18].

The success of fertility preservation emphasizes young patients’ masculinity and, therefore, their strength, promoting self-confidence in facing the disease and its burdensome treatments. In addition, it is reassuring for AYA patients to see that medical staff take their future into account, because it suggests that projection into afuture adulthood is possible.

However, some AYAs may not be able to collect sperm for several reasons. Firstly, a general deterioration in their condition can lead to such exhaustion that they are unable to take a semen sample even if they want to. In addition, in some cases, it may be the first approach to sexuality and masturbation. Another reason may be a state of stupor as a reaction to the diagnosis of such a serious and fatal disease. This state can occupy the entire field of thought with a deadly dimension and does not allow for the collection of sperm by masturbation. In such cases, psychological support is crucial to reduce emotional distress and allow for attention to be given to sperm collection [19]. Furthermore, sometimes, AYAs and their parents may be worried by a delay in the start of chemotherapy. Finally, a climate of despair and difficulties in seeing any positive element in the situation can increase doubts about the efficacy of fertility preservation techniques. When children and their parents are confronted with the risk of infertility associated with cancer and/or its treatment, their attitude can range from denial to catastrophism. Some may think that it does not matter, and the only thing that matters is to survive the disease. In contrast, others see the risk of sterility as an additional catastrophe. They project that this potential sterility will forever remind them that the disease managed to ruin their young adult lives. In this context, fertility preservation then appears as the possible preservation of a normal future. Young adult cancer survivors point out that the risk of infertility is of particular concern, and the ability to reuse their cryopreserved gametes seems to them like a passport to family life.

## 3. Perception of Sperm Freezing by Adolescents and Young Adults and Their Parents

Sperm freezing allows sperm to be preserved for years because no changes occur over time [20], even after more than 20 years of storage [21]. This is of particular interest to young pubertal boys. Some AYAs doubt the ability of spermatozoa to wake up from their “freezing sleep” and regain their original function as mobile cells capable of fertilizing an oocyte, thus allowing the cured patient to take the greatest revenge on the disease, i.e., to give life. They will be able to pass on this precious and fragile life that they themselves almost lost. Aware of the preciousness and fragility of life, cancer survivors who have become parents thanks to the reuse of their frozen/warmed spermatozoa are members of various patient associations. Some of them share their experiences on the internet, encouraging new young patients to preserve their fertility. There is a large body of research on fertility preservation in the care of young cancer patients. All guidelines agree on the importance of fertility preservation. Fertility counselling for AYA patients with cancer, regardless of their prognosis, is very important, with few expressing concerns about false hope or wastefulness, although most encountered barriers due to the patient’s medical status, treatment history, or even the medical team [22]. A recent review of the literature focused on the factors that promote and limit the decisions of young people and their families to pursue fertility preservation [19]. Of all the parameters considered in boys with cancer, pubertal status is the main facilitator, while non-heterosexual orientation and religion are exclusive barriers. The cost of fertility preservation can be an additional barrier, and relationship status is considered a barrier only from the perspective of some healthcare providers. Among patient attitudes, emotional distress and limited receptivity, not wanting children, no or little interest in fertility and fertility preservation, and religious beliefs may be the source of additional barriers to discussing fertility. For some patients, fear of passing on a genetic risk for cancer or fear of cancer recurrence and a lack of familial or social support are additional barriers [19].

Barriers to providers’ attitudes include a lack of time to discuss fertility, priority given to starting treatment, and a lack of documentation for patients. They can also have significant reservations about their lack of knowledge about fertility issues, preservation options, and the efficacy of assisted reproductive technologies [19].

However, only about half of AYA cancer patients actually pursue fertility preservation [19]. While some factors, such as general condition, can only be changed to a limited extent, the ease/difficulty for healthcare providers to raise the issue of fertility with cancer patients and their parents can be facilitated by the availability of supportive documentation (paper or virtual). For pubescent teenagers in the 21st century who are fans of YouTube, which allows access to knowledge through video documentation on any subject, the medium of video seems appropriate. To respond to the doubts of these young boys/men exhausted by the disease and to encourage them in their efforts to preserve their fertility, Video S1 was created, showing the awakening of warmed sperm after several years of freezing—in a few minutes, the spermatozoa regain motility on the spot and then progressively. This kind of support can promote the adherence of young boys and their parents to the fertility preservation process [19].

### 3.1. Representation of Sperm Cryopreservation: An Echo of Fairytales Symbolism

The impact of cancer on young patients is a traumatic event that leads to pain, hospitalization, and disruption of the continuity of daily life. It is difficult to find a way to communicate in such an intimate situation. For those who manage to obtain sperm by masturbation before gonadotoxic treatments, fertility preservation can be reassuring. This may suggest that the following stages, those of the grueling treatment, will also be successful. For those who fail to obtain sperm, feelings of weakness and inferiority are heightened. AYAs doubt their ability to emerge triumphant from the exhausting treatment that lies ahead. This underlines the importance of combining the fertility preservation process with additional psychological support.

The use of freezing as a preservation technique is not trivial. Some experiments, such as the creation of fairytale groups, have allowed onco-hematological children to recount and share their experience of illness in a different way, allowing them to symbolically express their pain [23].

As metaphorical narratives, tales have the symbolic function of representing the unconscious [24], allowing children and older people to translate the unspeakable, especially fears related to illness, sexuality, and the future. The falling asleep and waking up of sperm can be interpreted as a metaphor for the repression of potentiality, a suspended time that echoes the urge to live put on hold by illness. Falling asleep evokes a withdrawal, a resting of the vital forces (libido and life drive). Waking up symbolizes hope, the return of desire, the promise of the future, and, therefore, resilience.

Tales can aid in the elaboration of psychological conflicts [24]. This is particularly true for adolescents suffering from cancer, when the disease disrupts the process of adolescence—separation–individuation, the construction of sexual identity, and projection into the future. Tales make it possible to stage inner conflicts, especially those linked to symbolic castration (fear of losing one’s masculinity and fertility), and to sublimate the fear of death through a narrative that gives meaning, continuity, and controlled temporality.

Tales have also a containing and transitional function—stories act as a transitional object in Winnicott’s sense [25]; they provide an intermediate space between external reality and the inner world, allowing young patients to symbolically appropriate the medical experience by limiting its total invasion. They create a reassuring psychic distance and support the processes of symbolization that are undermined by the brutal eruption of a traumatic reality such as cancer.

Finally, tales have a restorative and narcissistic function—the preservation of fertility is linked to body image, virile power, and the ability for transmission. From a psychoanalytic perspective, tales can support a narcissistic restoration (“I am still capable”, I am still a sexual subject and potentially a father), as well as a symbolic recognition of trauma, without crushing it under the weight of raw reality (medical technology, fear, shame, etc.).

**Figure 1:**
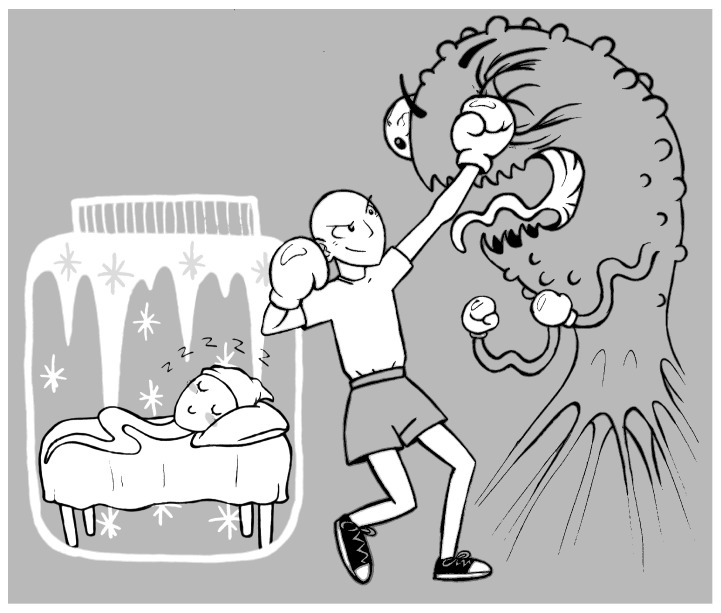
While the young patient fights cancer like a superhero, Sleeping Spermatozoon lies comfortably in its little ice straw. In the imagination of sick children, as in the imagination of everyone else, sleep plays a pivotal role in the overall plot of many fairy tales [27]. It can be experienced as a fantastic, mystical event that allows for a miraculous change in the course of history. For sick children, the underlying message may be that “sleep” is powerful and will help them and their cells to escape mortal danger.

Tales stimulate children’s imagination and help them to become aware of their difficulties by suggesting possible solutions to the problems they face [26]. The germ cells, like the heroes of fairytales, are “asleep” while terrible events take place (Figure 1) and the forces of evil are unleashed, as in *Sleeping Beauty* or in *Snow White*. These stories told to children are highly symbolic. The heroes will find themselves as victims, a position from which they will try to escape by dangerous means. The ending may or may not be happy. In some folk tales, the cessation of active life through sleep occurs at a time of maximum stress, and the resurrection of the heroine is followed by an advantageous reparation, since it is accompanied by a progression in maturity (*Snow White* or *Sleeping Beauty*). The evocation of death is often “softened” for children by the metaphor of a deep sleep. In the tale of *Snow White*, for example, on the following three occasions, the young girl loses consciousness three times (like the three drops of blood from which her name derives): once by being strangled by a ribbon, once by being poisoned by a comb, and finally, more seriously, by being poisoned by an apple. Inanimate and cold, she is left for dead by the dwarves and placed in a glass coffin to preserve her body. An external action (by Prince Charming) will wake her up, already a young woman. The happy ending of this story is completed by a wedding and many children. Freezing immobilizes sperm and allows them to remain dormant for as long as necessary to create the right conditions for their awakening, which can lead to life.

The same symbolism can be found in the tale of *Sleeping Beauty*—condemned by the evil fairy Maleficent, Aurora is saved from death by a good fairy who puts her into a deep sleep, during which the forces of evil are unleashed in the form of brambles for almost 100 years. Finally, Prince Charming, fighting with all his might against the sharp and very hurtful brambles that surround the castle where the girl rests, manages to defeat Maleficent’s forces, crosses the thick barrier of brambles, and brings the girl back to life with a kiss. The end of the tale is similar to the previous one, with many children for Aurora and her Prince Charming. In these two examples, sleep is assimilated with the context of “almost dead” but with the potential to return to an active, happy, and procreative life. Thus, these two fables evoke awakening and the return to life, a symbol that can also be used to illustrate the warming of sperm (Video S1).

### 3.2. The Particularity of the Potential Use for Posthumous Procreation

The possibility of preserving samples after death is available in some countries and raises many questions for patients and especially their families. The potential use of posthumous sperm from AYAs provides bereaved parents and/or partners with a sense of transcendence of death or immortality, both literally and symbolically. The possibility of posthumous reproduction for a cancer patient with a poor prognosis presents an ethical challenge. The storage of gametes can represent hope for the family left behind in the event of death [28]. Thus, the possibility of preserving something of the deceased child and having the chance to use it to continue the family lineage to another generation is a unique way to restore self-esteem and a sense of value and meaning. The prolonged existence of one’s children may also act as a buffer against fear of death. That is, offspring may be perceived as an accessible means of attaining symbolic immortality, as the self is extended to a generative organism. In addition, this organism is perceived as being physiologically and psychologically similar to the individual self, and, thus, being a good symbolic representative of the individual self [17]. However, there are ethical, legal, and societal discrepancies worldwide [16]. Societies such as Israel, which emphasize the importance of family and the value of having children, often see parenthood as vital to the long-term strength of the group, making it particularly difficult to cope with the death of a child [16]. In Islamic countries, posthumous procreation is prohibited because assisted reproductive technologies are only allowed if both parents are alive [29]. In the United States, posthumous gamete retrieval or use for reproductive purposes is ethically justifiable if there is written documentation from the deceased person authorizing the procedure [30]. The posthumous use of frozen sperm is prohibited in France.

## 4. The Impact of Cancer and Its Treatment on Sperm Quality in AYAs

An important piece of information, which is more technical but essential, concerns the biological and technical impacts of AYA sperm freezing. Since the first experiments by Lazaro Spallanzani in 1776, sperm freezing has improved significantly. In 1953, Bunge and Sherman published the first births after insemination with human sperm frozen in dry ice [31]. The use of liquid nitrogen for long-term storage allowed the development of sperm banks [32], paving the way to the preservation of male fertility in the context of cancer. The usefulness of frozen sperm prior to oncologic treatment is confirmed by the consistent proportion of men who use frozen sperm despite not being azoospermic [33]. This already old technology plays an important role in the supportive care of several serious diseases such as cancer. Sperm quality after thawing can be affected by the patient’s pathology, as well as some technical parameters such as cryoprotectants, ice formation, storage conditions, and osmotic stress during the freezing/thawing process. In cancer, the motility and viability of thawed sperm depend on the basal semen quality and tend to be reduced. However, the impairment of these parameters does not seem to be related to the age of the patient or the duration of cryopreservation [34]. In sperm, high levels of oxidative stress may affect the survival of frozen sperm (see below), increase sensitivity to cancer treatments, and alter the sperm epigenome. In the case of testicular cancer, which is the most common solid tumor in AYAs, sperm parameters are more impaired than in other malignancies. Furthermore, the impact also varies according to the age of the patient, the histological type of the tumor, and the history of previous orchidectomy [35,36,37,38,39]. This use may be associated with a significant change in sperm parameters, the impairment of sexual ability, or ejaculatory dysfunction. There is also a hypothesis that chemotherapy may cause some irreversible DNA damage or epigenetic modifications that are transmitted to offspring [40]. In the case of lymphomas, the second most common malignancy in young men, changes in sperm parameters appear to be inconsistent [41,42,43]. When reported, the severity of changes correlates with the stage of the disease [41]. Sperm volume, vitality, and number are reduced [42]. These abnormalities could be the consequence of fever and night sweats, as well as immune-mediated disorders in patients with Hodgkin’s lymphoma. Furthermore, spermatozoa from patients with lymphoma have a higher rate of aneuploidy compared with healthy patients, regardless of whether the lymphoma is Hodgkin’s or non-Hodgkin’s [42,44]. The imminent mortal danger for germ cells can be perceived by children/adolescents as an echo of the imminent mortality of cancer. In addition, counseling on the risk of sterility points to the danger of targeting very specific cells which are not essential for survival but which promise a return to a normal life, with the possibility of starting a family and having children. The possibility of saving reproductive cells should not be at the expense of reducing the chances of eradicating the disease. For adolescent boys, the possibility of preserving their fertility by freezing their sperm should be systematically offered whenever reprotoxic treatment is considered. AYA patients are invited to project themselves into a future where they can play the role of an adult who wants to have a child. For them, this means the possibility of healing and of returning to a normal, happy life.

## 5. Conclusions

When AYAs are confronted with a serious illness such as cancer, they become more or less aware of the threat of death hanging over them. Throughout treatment, the impact of the disease and its treatments will require AYAs to fight the disease like a hero. Echoing this, cured children are called cancer survivors, just like survivors of a major war, and they may like to feel (and dress up) like superheroes. As an illustration, many children with cancer can be seen in the corridors of pediatric oncology departments around the world wearing superhero costumes, and AYAs often wear T-shirts depicting superheroes. Years later, the period they lived through is a painful memory that many find difficult to evoke. In this period of treatment, psychological determination and courage are supported by family and caregivers, who promise the child that difficult treatment is the way to recovery. In the collective unconscious, including in children, sleep is a process that helps us to survive extreme situations. In the case of anesthesia, sleep prevents the experience of particularly intense pain and makes life-saving surgery possible. Freezing sleeping can allow reproductive cells to survive life-threatening danger. From a clinical point of view, information on sperm cryopreservation may integrate the symbolic dimension of this approach. We propose the concept of “sleeping spermatozoa”, illustrated by a video, which is, today, the communication support used by new generations.

## Data Availability

Not applicable.

## Supplementary Materials

The following supporting information can be downloaded at: https://www.mdpi.com/article/10.3390/life15050685/s1, Video S1: Waking up Sleeping Spermatozoa.
